# Supplementary material for: Hedgehog dysregulation contributes to tissue-specific inflammaging of resident macrophages
Source: Aging (Albany NY). 2021 Aug 14;13(15):19207–29. doi: 10.18632/aging.203422 (PMC8386529; doi:10.18632/aging.203422)
Supplement: Supplementary Figures [file aging-13-203422-s001.pdf]

SUPPLEMENTARY FIGURES

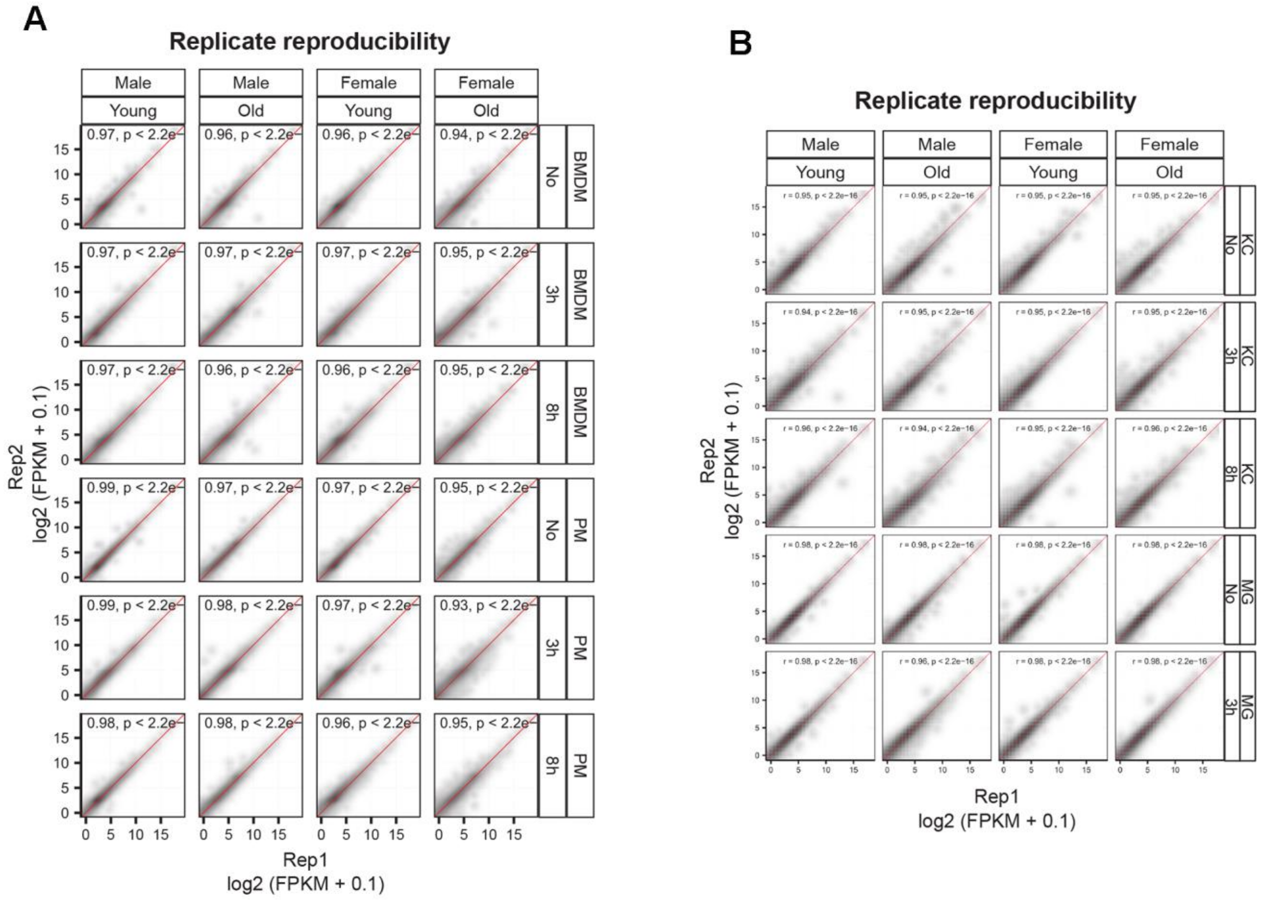

Supplementary Figure 1. Reproducibility of total RNA-seq between biological replicates. (**A**) BMDM and peritoneal macrophage (PM) samples. (**B**) Kupffer cells (KC) and microglia (MG) samples. Rep1: replicate 1, Rep2: replicate 2. Numbers inside each plot are Pearson correlation coefficients and p values.

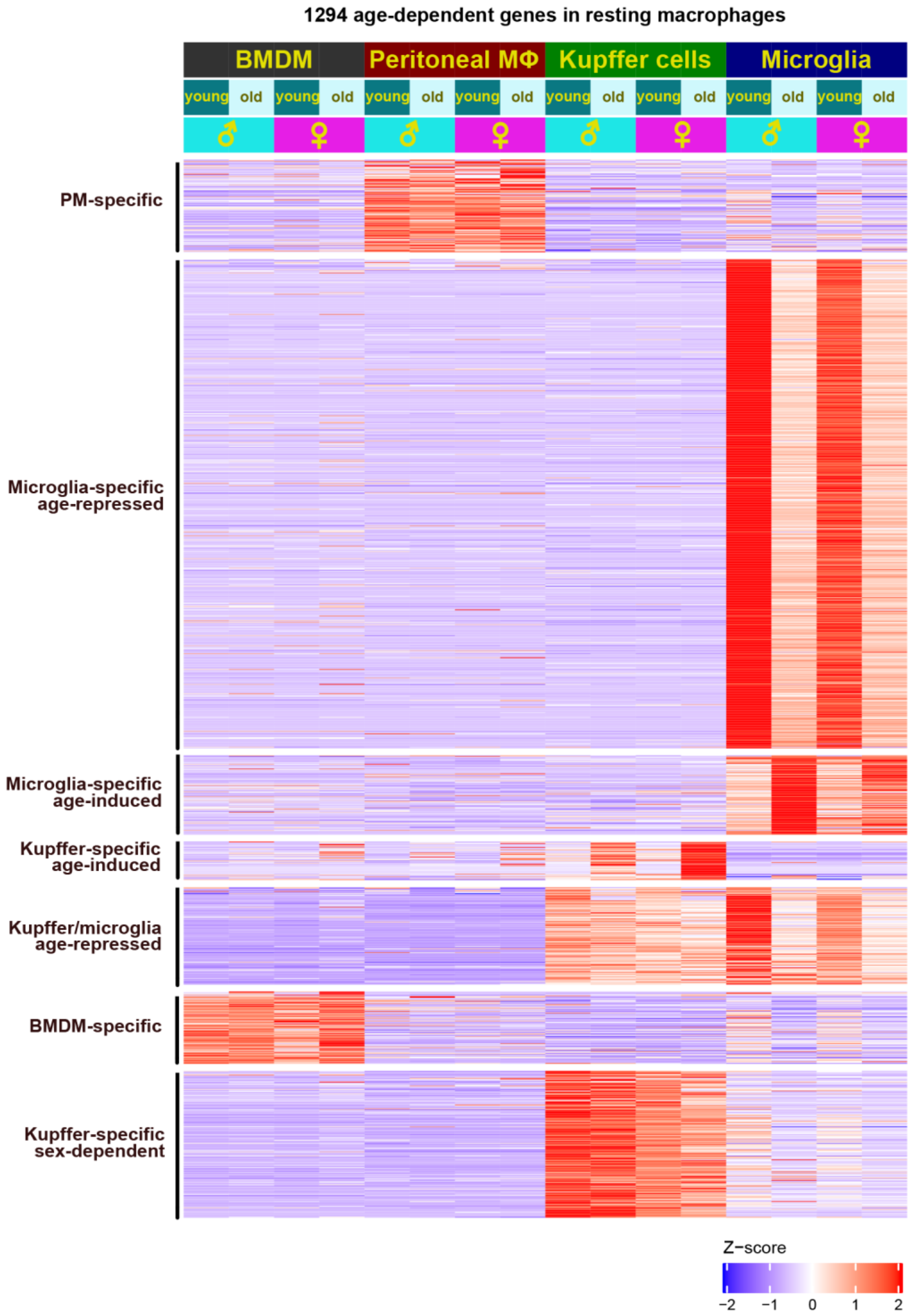

Supplementary Figure 2. Age-related changes in the basal transcriptome of tissue-resident macrophages. The heatmap shows the result from a cluster analysis of genes whose basal expression were age-dependent. The descriptions of genes in the clusters are provided in supplementary Table 2.

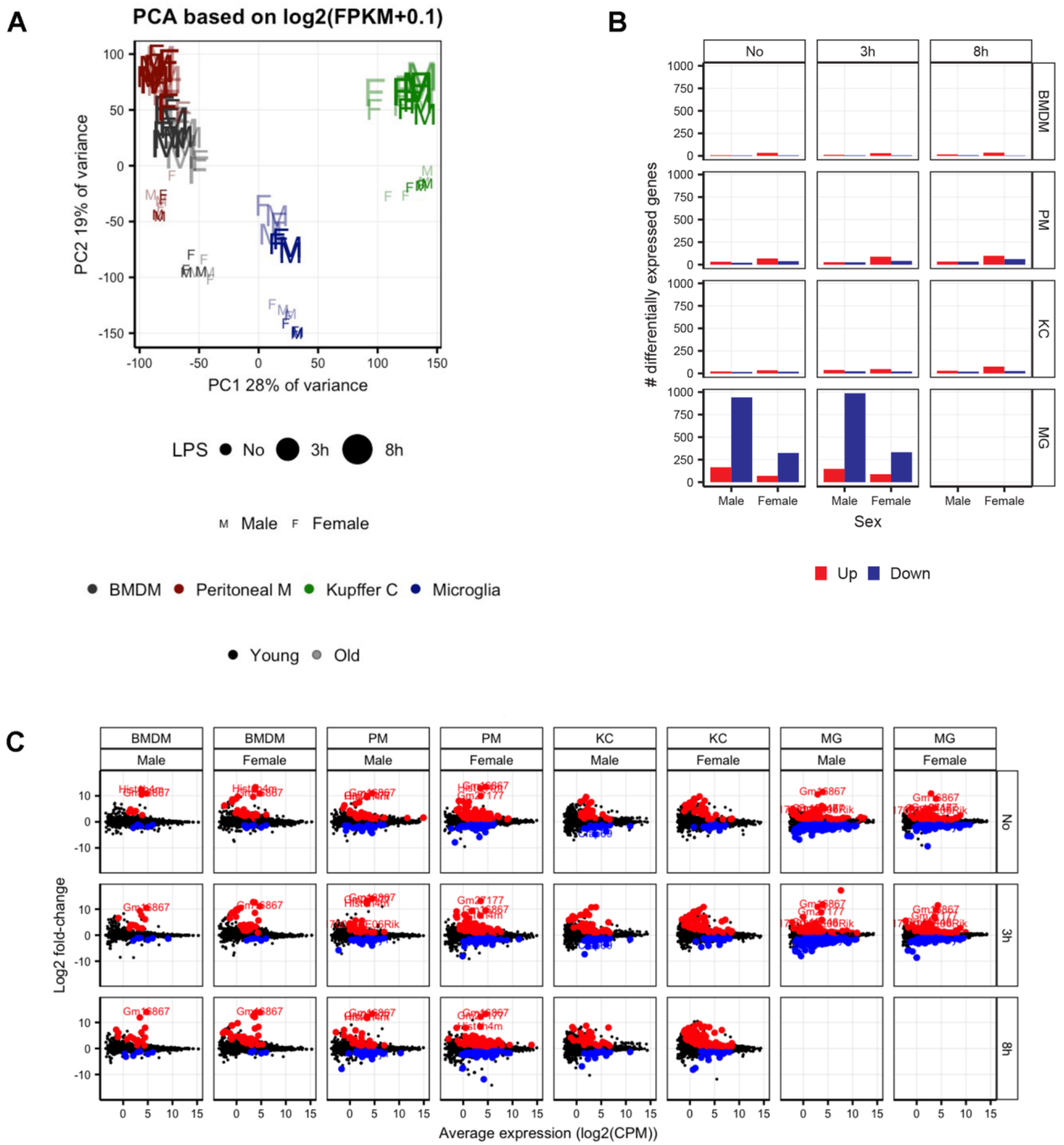

Supplementary Figure 3. Tissue-specific changes in aging macrophage populations. (**A**) PCA plot of four macrophage populations along LPS time course and in aging. (**B**) Number of differentially expressed genes between age groups in pairwise comparisons performed separately for each of the four macrophage populations and for each stimulation condition and sex. (**C**) MA plots show RNA-seq data in log ratios of old / young values for the indicated sample group. CPM, count per million reads. BMDM: Bone marrow-derived macrophages, PM: peritoneal macrophages, KC: Kupffer cells, MG: microglia. LPS 8h condition was not sampled for microglia due to insufficient number of cells. Red: up-regulated in old, Blue: down-regulated in old.

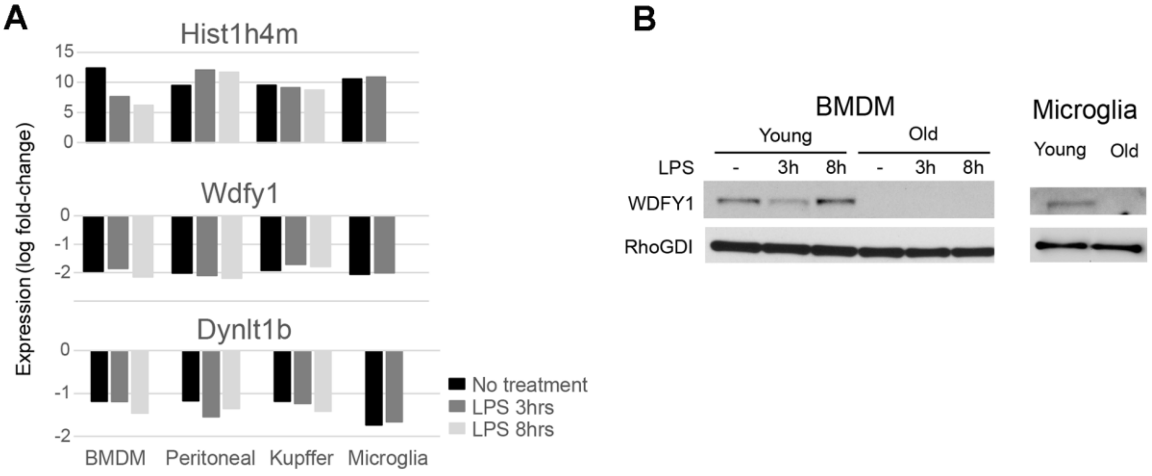

Supplementary Figure 4. Genes commonly dysregulated with age across the four macrophage populations and all stimulation conditions. (**A**) RNA-seq expression data are shown in logratios of Old / Young FPKM values. (**B**) Western blot shows WDFY1 protein expression in BMDM and microglia from young or old mice.

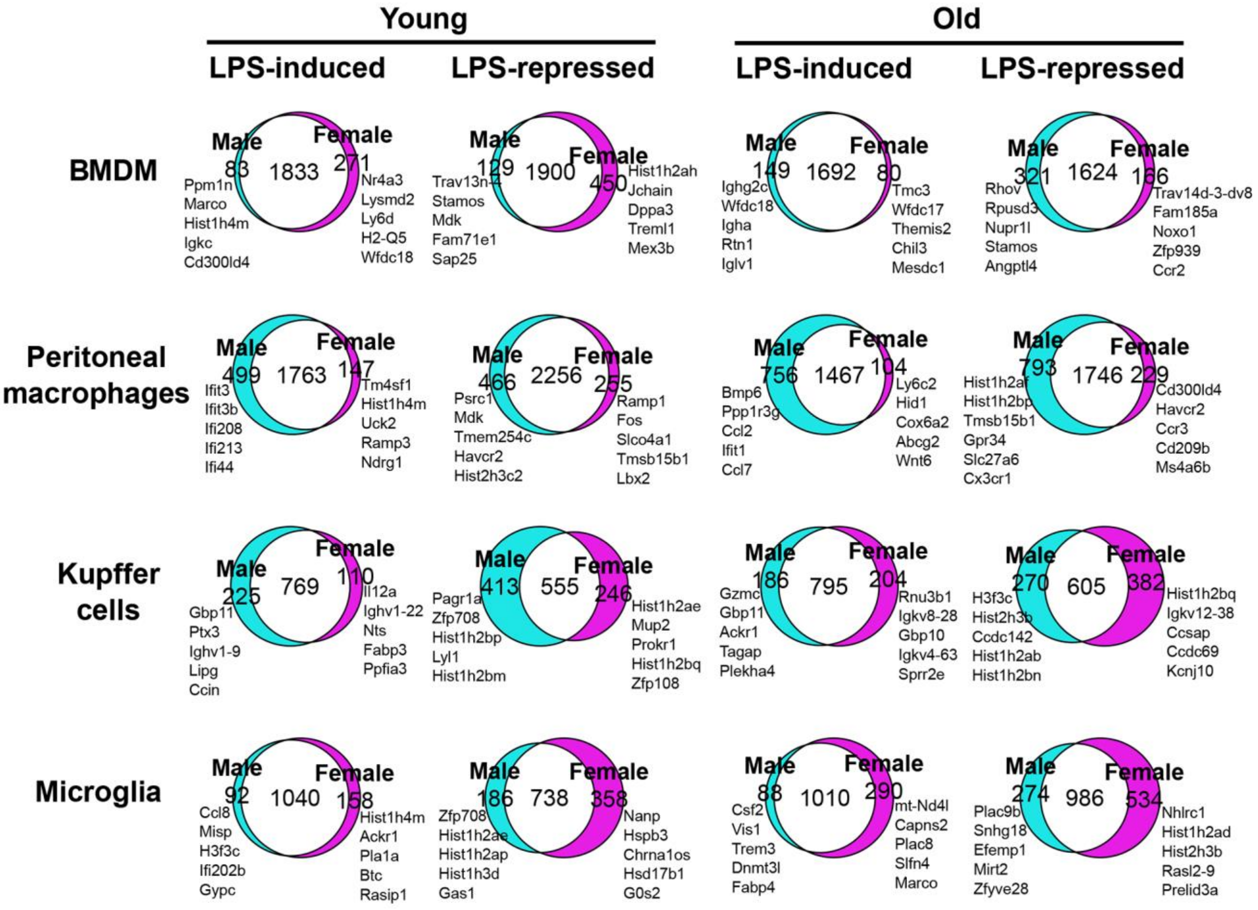

Supplementary Figure 5. Age-associated sex differences in endotoxin responses of tissue-residence macrophages. Venn diagrams show the numbers of LPS-regulated genes common and unique to male and female mice. Each set of LPS-induced or -repressed genes was defined as those differentially expressed between macrophages untreated and treated with LPS (10 ng/ml) for 3 hours. The names of top genes based on 3h fold change +/- LPS are shown next to each Venn diagram. The areas of Venn diagram regions are proportional to the size of the represented subsets.

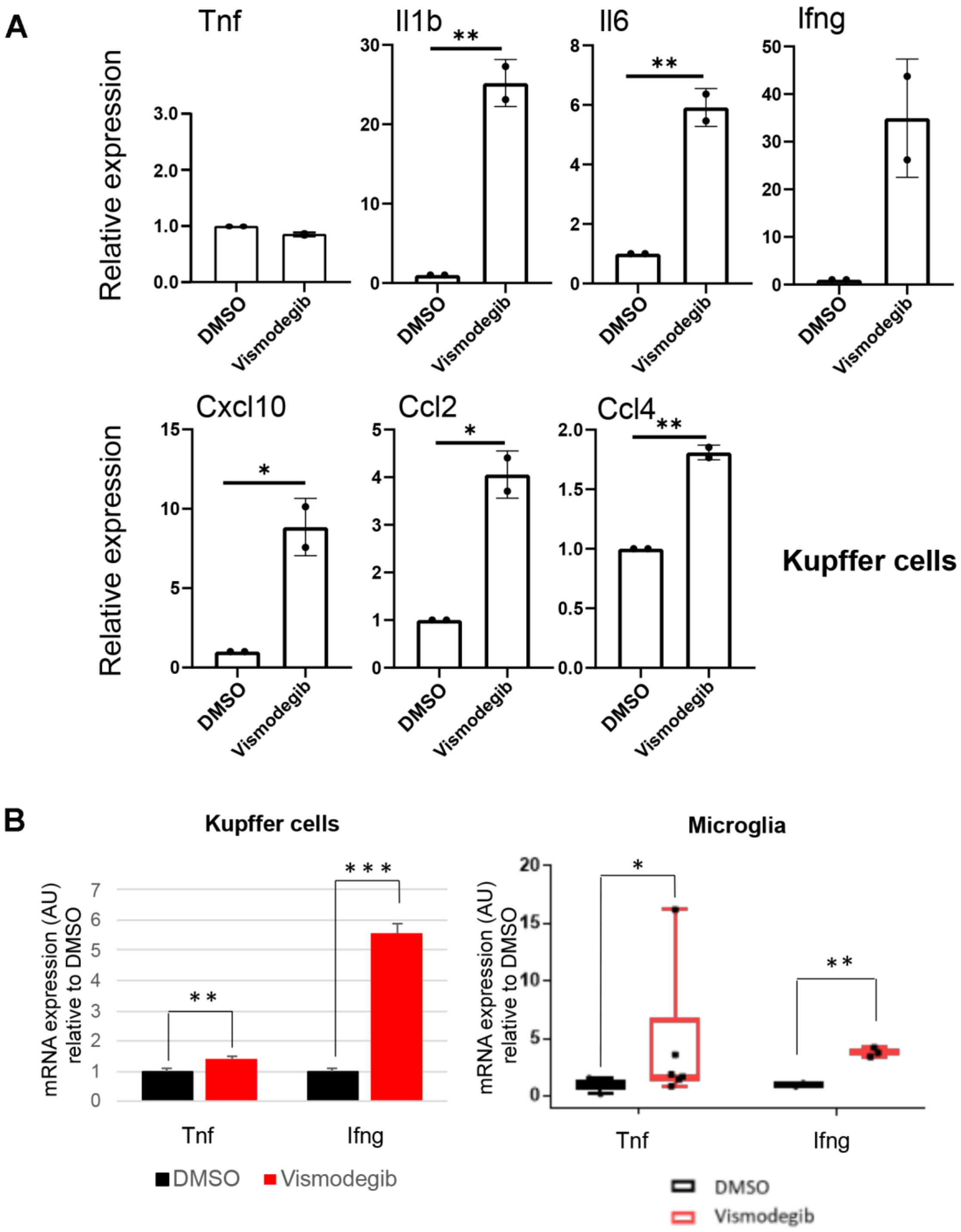

Supplementary Figure 6. Reduction of hedgehog signaling promotes the expression of pro-inflammatory cytokines in tissue-resident macrophages. (**A**) Young mice (2-3 months old) were injected intraperitoneally with vismodegib (75 mg/kg) or DMSO, and RNA was subsequently extracted 1 hour later from Kupffer cells. RT-qPCR was performed to measure the expression of indicated transcripts. mRNA expression levels were normalized to Actb (beta actin) and are shown relative to DMSO treated animals. Representative data from 3 biological replicates are shown. (**B**) Independent biological replicates of data in (**A**) and data in Figure 5A for Tnf and Ifng transcripts. mRNA expression levels were normalized to Gapdh and are shown relative to DMSO treated animals. Error bars: s.d. Asterisks indicate Student's T test p values (*: < 0.05, **: < 0.01).

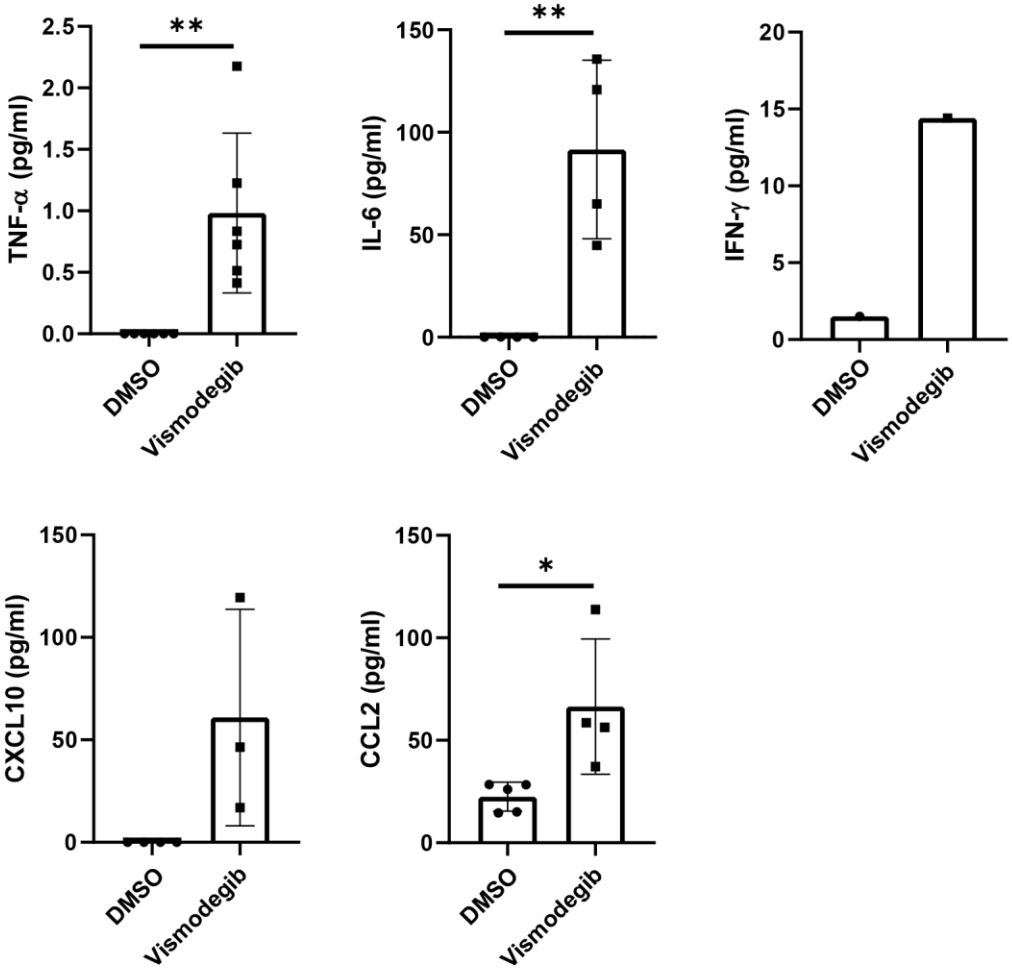

Supplementary Figure 7. Reduction of hedgehog signaling elevates the levels of pro-inflammatory cytokines in circulation. Young male mice (2-3 months old) were injected intraperitoneally with vismodegib (75 mg/ kg) or DMSO, and serum was subsequently collected one hour later from six animals per group. ELISA was performed to measure the concentrations of indicated cytokines. Data are from two biological replicates, each with technical replicates. Error bars: s.d. Asterisks indicate Student's T test p values (*: < 0.05, **: < 0.01) when there were sufficient replicates. Wells with undetectable reading were given zero values.
